# Implementation of Brain Breaks^®^ in the Classroom and Its Effects on Attitudes towards Physical Activity in a Chinese School Setting

**DOI:** 10.3390/ijerph18010272

**Published:** 2021-01-01

**Authors:** Ke Zhou, Sensen He, Yanli Zhou, Biljana Popeska, Garry Kuan, Li Chen, Ming-Kai Chin, Magdalena Mo Ching Mok, Christopher R. Edginton, Ian Culpan, J. Larry Durstine

**Affiliations:** 1 Sports Reform and Development Research Center of Henan University, School of Physical Education, Henan University, Kaifeng 475001, China; zhouke221@126.com (K.Z.); zhouyl1977@126.com (Y.Z.); 2 Minsheng College, Division of Henan University, Kaifeng 475001, China; hss1109@126.com; 3 Faculty of Educational Sciences, Goce Delcev University, Stip 2000, North Macedonia; 4 Exercise and Sports Science Programme, School of Health Sciences, Universiti Sains Malaysia, Kubang Kerian 16150, Malaysia; garry@usm.my; 5 School of Physical Education and Health Science, Chongqing Normal University, Chongqing 401331, China; swuchenli@163.com; 6 The Foundation for Global Community Health, 1550 W Horizon Ridge Pkwy Ste R #206, Henderson, NV 89012, USA; mingkai@gchfoundation.org; 7 Graduate Institute of Educational Information and Measurement, National Taichung University of Education, 140 Minsheng Road, West District, Taichung City 40306, Taiwan; mmcmok@friends.eduhk.hk; 8 Assessment Research Centre, Department of Psychology, The Education University of Hong Kong, 10 Lo Ping Road, Taipo, N.T., Hong Kong; 9 Human Performance Center, University of Northern Iowa, 105, Cedar Falls, IA 50614, USA; christopher.edginton@uni.edu; 10 School of Health Sciences, University of Canterbury, Private Bag 4800, Christchurch 8140, New Zealand; ian.culpan@canterbury.ac.nz; 11 Department of Exercise Science, University of South Carolina, Columbia, SC 29208, USA; ldurstin@mailbox.sc.edu

**Keywords:** children, video exercises, physical activity, attitudes, online platform, Brain Breaks^®^, classroom-based intervention

## Abstract

This study aimed to examine the effects of three-months of classroom-based Brain Breaks Physical Activity Solution (Brain Breaks^®^) on attitudes toward physical activity levels of primary school children in Henan Province, China. The participants were 704 children enrolled in grades 3–5 who were randomly assigned to either an experimental or a control group. The experimental group participated in Brain Breaks^®^ video intervention for 3–5 min daily, at low-to-moderate intensity for three consecutive months. The control group resumed their normal routine. The children’s attitudes in both groups towards physical activity (PA) were evaluated using the self-reported Attitudes toward Physical Activity Scale (APAS), before and after the intervention. The effects of the intervention on *APAS* scores were analyzed using mixed model analysis of variance with Time as within-subject, and Group as between-subject factors. The analysis revealed evidence in support of the positive effect of classroom video interventions such as *Brain Break* on children’s attitudes toward importance, fun, and trying to do their personal best in physical activity. Also revealed was the important role the teacher plays in this process.

## 1. Introduction

A healthy active lifestyle and one’s overall well-being are crucial to achieving a good quality, happy, and fulfilled life. As one of the elements of healthy living, physical activity (PA) has many short and long-term health benefits across all ages. When considering children, PA is important for proper motor development [1,2], physical fitness [3] and the development of movement habits [4]. PA also improves cognitive function, ability to maintain attention, developing and maintaining a working memory, cognitive flexibility [5,6,7,8] brain functioning [9,10], academic performance [3,6,11,12,13], cognitive ability [8], emotional reactions [14], social engagement, and mental health [3,9,13]. Cross-sectional studies provide strong evidence for an association between complex motor skills and cognitive skills in developing children, especially in the prepubertal period (under 13 years) [7,15]. Furthermore, low levels of PA engagement are associated with uncontrollable emotions [16]. Biddle et al. [9] found evidence that higher PA and fitness levels are associated with improved motor performance, cognitive health, cognitive functioning, and self-esteem.

Many different internal and external factors influence children’s PA levels. A sedentary lifestyle, a lack of available time, an over-crowded curriculum, a lack of interest and skills, and prolonged screen time are some of the internal factors identified as responsible for low PA levels [4,6]. Family, school, community, governmental policy, environmental factors, and the contemporary manner of one’s living circumstances are examples of external factors that positively and negatively affect children’s PA levels [6]. The World Health Organization (WHO), the leading organization for the promotion of health and PA, launched the Global Action Plan on Physical Activity 2018–2030 [17] as a framework for effective policies and actions aimed at promoting global PA. This plan emphasizes cooperation in health, sport, transportation, urban design, academia, and civil society to promote PA. Furthermore, the aim of increasing PA directly contributes to the United Nations Sustainable Development Goals (SDG) [18], particularly to SDG#3 (good health and well-being). Other goals, including SDG#4-quality education, referring to quality Physical Education (PE) and PA interventions in schools, SDG#11-sustainable cities and communities, aiming to raise attention concerning community actions for promotion of PA, and active transportation make important contributions. The efforts of the WHO in alliance with the International Olympic Committee to promote health through PA and sport contributes to the prevention of non-communicable diseases, especially in the context of the COVID-19 pandemic [19].

Despite initiatives to increase PA levels, many parts of the world remain below recommended levels. For children aged 5–17 years, the WHO (2010) recommends at least 60 min of daily moderate-to-vigorous intensity PA [20]. Activities could take the form of play, games, sports, active transportation, recreation, PE classes, and other aerobic activities that integrate movements to strengthen muscles and bones [20]. Notwithstanding, global trends have shown decreasing PA levels among children alongside increased health risks [21,22,23,24,25]. Only a small number of children are able to meet the minimum criteria for PA as recommended by the WHO [26,27]. Reasons for low PA levels include poor eating habits [28], sedentary behavior [29,30], longer screen time, the use of technology associated with longer sitting time [23,31,32], and excessive use of motor vehicles [33]. Similar trends towards reduced PA are noted among Chinese children. Liu et al. (2018) [34] using China’s PA report card found low PA levels among 9 to 17-year-old children. Zhu et al. [35] reported contrary results and found a small increase in PA trend among Chinese children (29.9% in 2016 to 34.1% in 2017). In addition, a barrier preventing Chinese children and youth from exercising is the high level of China’s air pollution [36,37]. Other environmental conditions such as rain and snow often restrict Chinese children from performing PA. A huge population and scarce resources make the number of school sporting venues insufficient for children to conduct daily PA, which also contributes to children’s limited PA participation [38].

Benefits of regular PA for health and overall development, on the one hand, and reports of decreased PA levels among children for numerous reasons, on the other hand, give rise to two important questions: (1) How can children be motivated to be physically active on a regular basis and (2) How can the pitfalls of modern technologies such as televisions, computers, laptops, tablets, smartphones, and wearable devices be exploited to initiate PA? Solutions are found in school and classroom-based PA interventions supported by technology that strive to increase children’s daily PA. Such initiatives are applied in addition to PE classes and are not a PE replacement but are complementary to school PE.

Children are more likely to participate in classroom activities that are interesting, fun, and enjoyable. Interactive video games, a variety of mobile applications, and internet-based PA interventions do stimulate children’s PA interest and promote participation in an active lifestyle [39,40]. Applied correctly, technology is fun, interactive, an effective pedagogical strategy for learning and an effective tool for motivating movement [41]. With the current COVID-19 pandemic, technology-supported PA is often recommended as a solution to cope with associated negative pandemic effects. Use of mobile applications, online video exercises, short sitting breaks, and self-monitoring training applications are means for improving PA during the pandemic [42,43].

Developing daily classroom-based PA routines with the full use of technology is an integrated pedagogical approach that promotes children being physically active. A promising intervention has been developed by HOPSports, named Brain Breaks^®^ Physical Activity Solutions (Brain Breaks^®^) [44]. Classroom-based and technology-supported interventions integrate different types of movement in 3–5-minute online videos, aimed to enhance PA during theoretical lessons, and to improve children’s interest in learning and well-being. Online exercises are specifically designed to motivate children to become physically active, to learn new motor skills, languages, art, music, and to become knowledgeable about different cultures [45]. The effects of Brain Breaks^®^ are confirmed in many studies conducted with primary school children in more than 15 countries [46,47,48,49,50,51,52,53,54,55]. Positive *Brain Breaks*^®^ intervention effects include improved PA, improved self-efficacy in learning with the use of video exercises [46,47], improved self-awareness and self-confidence [47], improved attitudes toward PA [48,49,50], improved short-term memory [51], improved knowledge of PA as a health-related behavior [49], improved goal orientation and holistic learning [4,46,47,48,52,53], augmented PA behavior change, improved cognitive and behavioral processing, and improved internal feelings [54]. Recently, Brain Breaks^®^ was found to enhance motivation toward PA in Type-2 diabetes mellitus patients [55] and as a solution for inactivity due to the COVID 19 pandemic [56].

In the recent China Education Modernization 2035 document issued by the Central Committee of the Communist Party of China and the State Council [57], ten main development goals for advancing and modernizing education by 2035 are presented. Within these development goals, Goal 5 is focused on building a holistic person through a flexible lifelong learning system; Goal 7 is intended to build a team of high-quality professional and innovative teachers; Goal 8 aims at using modern technology to accelerate individualized learning and sharing of digital educational resources; and Goal 9 is intended to improve and enhance international cooperation and faculty exchanges to gain experience of mutual learning between countries [57]. Despite the positive experiences and benefits of implementing Brain Breaks^®^ intervention, no study was found in the literature incorporating Brain Breaks^®^ in Chinese classrooms. Thus, an intervention was developed and applied to 3rd to 5th-grade primary school children in China’s Henan province. The study aimed to investigate the effects of a three-month classroom-based intervention on attitude toward PA. The main study hypothesis is that participation in Brain Breaks^®^ performed on a daily basis and implemented for three consecutive months will change attitudes toward PA in Chinese school children.

## 2. Materials and Methods

### 2.1. Study Design, Recruitment and Sampling

This study employed a quasi-randomized experimental research design. Two schools were selected based on the approval from the Ministry of Education in the Henan province, China. Prior to intervention, trained research assistants visited each school and introduced the study procedure to administrators and faculty, and trained teachers to use the intervention digital platform provided by HOPSports. Intervention was conducted daily for three consecutive months. Teachers selected from various 3–5-min children’s videos each day, with an accumulated time of 30 min per week. The intensity of the exercises ranged from low to moderate and included exercise videos from across the world, such as Greek Zorba dance, Malaysian Silat, Italian hip-hop, wushu, taekwondo and many more. Teachers also complete daily and monthly reports showing the number of videos utilized per month. Before and after implementing the program, data on children’s PA behavior and self-reported participation in the program were collected and analyzed. The demographic information and the Attitude towards Physical Activity Scale (APAS) were numbered and coded. The control group did not complete an intervention but did complete the questionnaire survey at the same time as the experimental group.

### 2.2. Participants

Participants included 704 primary school children (370 boys and 334 girls) from 3rd to 5th grade (Table 1). Primary school children from Henan province, China were recruited for the study. Children (*n* = 780) agreed to participate and complete questionnaires. The inclusion criteria for the present study were: (1) Chinese students studying aged 9–11 years old; (2) Ability to read and write in the Chinese language and answer the questionnaire; (3) Low-risk status, indicating freedom from medical conditions measured by the physical activity Readiness Questionnaire (PAR-Q); and (4) Having completed and returned the parental and assent forms. Students with prior injuries or medical conditions, such as heart problems, were excluded. In this study, seventy-six (*n* = 76) children were excluded from the analysis for not properly completing the questionnaire. Children (*n* = 704) were randomly assigned to either experimental/intervention (*n* = 353) or control groups (*n* = 351).

### 2.3. Instruments

The study employed a self-report instrument titled Attitudes toward Physical Activity Scale (APAS) developed and validated by Mok et al. [52]. The instrument was specifically designed to investigate children’s’ attitudes, beliefs, and self-efficacy toward PA. The APAS questionnaire consists of seven sections composed of different items related to aspects of PA engagement: (F1) Benefits: Promoting holistic health; (F2) Importance of exercise habits; (F3) Learning: Self-efficacy in learning with video exercises; (F4) Self-efficacy: Self-efficacy in using video exercises;(F5) Fun: Exercise motivation and enjoyment; (F6) Fitness: Self-confidence in physical fitness and (F7) Personal Best: Trying to do your personal best. Participants were asked to respond to each item of the seven sections using a four-point Likert response scale with response categories: Strongly Disagree, Disagree, Agree, and Strongly Agree. This scale’s reliability and validity were established as part of a larger project and applied in several countries including Lithuania [49], Poland [46], Turkey [48], Malaysia [51] and Macedonia [47].

The original English APAS questionnaire version was validated previously. Rasch analysis provided empirical support for reliability, unidimensional effectiveness of response categories, and absence of scale gender differential item functioning (DIF). Questionnaire items were translated from English to Chinese, and the translated version was adjusted for cultural appropriateness before being administered. Specifically, a bilingual translator blindly translated the APAS questionnaire along with the instructions, response categories, information sheet, and informed consent form from English to Mandarin. A second bilingual translator translated the material back from Mandarin to English. The two English versions of the materials were compared for equivalence and were reviewed and finalized by a panel of five experts with experience in sport sciences, sport psychology, health psychology, and PE. Panel members were bilingual and able to speak in Mandarin and English and had more than 10 years of working experience in their respective field of expertise. Differences were corrected, and the contents evaluated for cultural suitability. Ten individuals were invited to assess the clarity of the final Chinese version and were asked to respond to the items giving their views on the questionnaire’s contents and presentation. The Chinese translated version of APAS was found reliable based on the value of Cronbach’s alpha. The reliability analysis was performed using the pre-test data, and the results are presented in Table 2. The internal consistency based on Cronbach’s alpha value ranged from 0.65 to 0.76 which indicates acceptable reliability.

### 2.4. Ethics

All study procedures were reviewed and approved by the Henan University’s research review board. Study participants were volunteers and could discontinue participation at any time without penalty. Prior to study enrollment, all participants provided written informed assent in conjunction with parental written informed consent. Approval from the school’s principal was also acquired.

### 2.5. Statistical Analysis

The data were analyzed using statistical software with SPSS 25.0. Effects of applied Brain Breaks^®^ intervention on APAS scores were analyzed using a two-way 2 (Time: before or after intervention) × 2 (Group: experimental or Control) mixed analysis of variance (ANOVA) with Time as the within-subject factor (repeated measures) and Group as the between-subject factor. The partial eta-squared (*η*^2^) effect sizes for the tests were calculated to indicate the magnitude of the effects. The level of statistical significance was set as *p* < 0.05.

## 3. Results

Presented in Table 3 are the mean scores for the APAS obtained by the experimental and control groups before and after the intervention. Also presented in Table 3 are the results of the 2 × 2 mixed ANOVA with one within-subject factor (Time: before or after intervention) and one between-subject factor (Group: experimental or control), as well as the effect sizes (Time *η*^2^, Group *η*^2^ and Time * Group *η*^2^). Highlighted in Table 3 is a significant increase in the mean scores of all APAS scales obtained before and after the three-month intervention. The main Time effect was significant (*p* < 0.001) for all *APAS* scales. The effect sizes of Time for five of the scale were only small (*η*^2^ ranged from 0.05 for the Importance scale to 0.10 for the Benefit scale), and medium for the remaining two scales (*η*^2^ = 0.19 for the Fitness scale, and *η*^2^ = 0.18 for the Personal Best scale.)

Presented in Table 3 are the results showing significant main effects of Group for Learning (*p* = 0.009), Fun (*p* = 0.007), Fitness (*p* < 0.001), and Personal Best (*p* < 0.001) scales. Effect sizes for the Group main effect were small (*η*^2^ < 0.10) for all scales.

**Figure 1:**
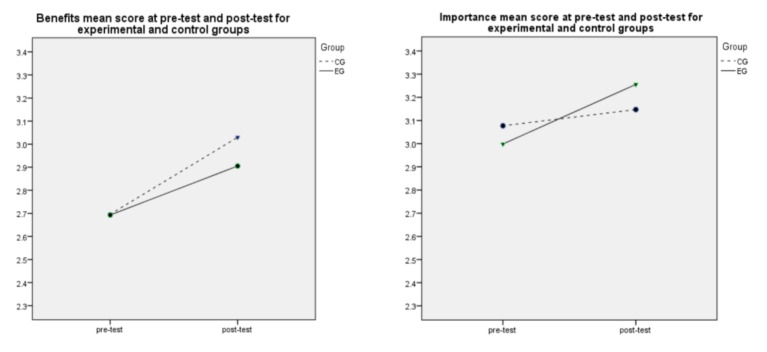

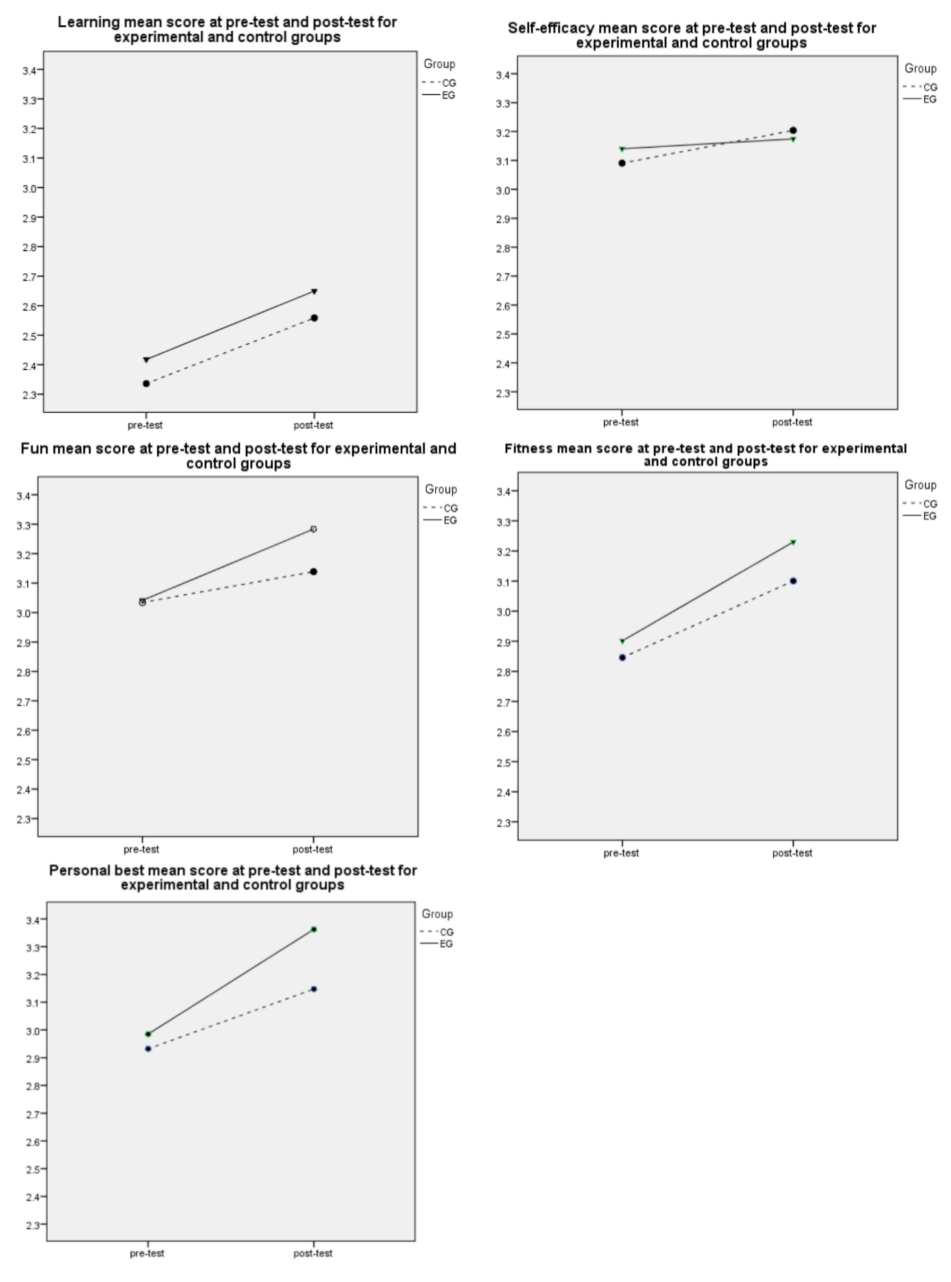
Scale Mean Values of the Experimental and the Control Groups at Pre-test and Post-test.

Changes across Time were not the same for three APAS scales of the two Groups, as indicated by the significance of the Time * Group interactions (Table 3). Time * Group interactions were significant for Benefits (*p* = 0.047), Importance (*p* = 0.001), Fun (*p* = 0.008), and Personal Best (*p* = 0.001) scales. The main values of experimental and control groups for each scale for pre-test and post-test are illustrated at Figure 1. As presented, at the baseline of the intervention, during pre-test time experimental and control group were similar in scores in every dimension. The slight differences in main scores were not statistically significant. Figure 1 displays that the experimental group increased more than the control group from pre-test to post-test in Importance, Fun, and Personal Best scales.

## 4. Discussion

This study evaluated the effects of classroom-based and technology-supported Brain Breaks^®^ on children’s attitudes toward PA after a three-month intervention. The study’s hypothesis was supported by the changes found in all seven APAS scales using a sample of Chinese children. Findings are similar to results from studies evaluating the effects of Brain Breaks^®^ intervention for attitudes toward PA [4,46,47,48,49,53].

Time and Group interaction effect (Time * Group *η*^2^) confirmed significant positive changes in four of seven APAS scales. Video exercise breaks significantly improved children’s understanding for being physically active across the lifespan (Importance of exercise habits). Experiencing fun and enjoyment during activity breaks was motivation for PA (Exercise motivation and enjoyment) and for improving personal best goal orientation (Trying to do personal best). In addition, recognizing the benefits of holistic health might also enable children to be inspired to perform PA (Promoting holistic health). Mok et al. [53] evaluated the effects of Brain Breaks^®^ on attitudes of children from eight countries (Croatia, Lithuania, Macedonia, Poland, Romania, Serbia, South Africa, and Turkey). Data from this study provides supporting evidence for our finding of the positive effects of enjoyment when engaging in PA.

Classroom-based PA interventions used in this study impacted the APAS scale for “Importance of exercise habits” and revealed that even small doses of PA during the school day are meaningful. Short in-school PA breaks lead to benefits and support the development of life-long PA habits. We argue, as do Martin et al. [58], that awareness of the importance of movement is the first step toward PA participation. Furthermore, the internal motivation for PA participation, independent of time and external environment, can engage children in actively taking part in exercise [58]. Given these findings, a multilevel approach combining curricular education during classes with non-curricular education during short breaks [48] does yield a promising result in the development of PA habits. This finding contributes to children’s overall holistic development and aligns with findings associated with studies by Aubert et al. [23] and Wang et al. [38].

The evidence provided by this study supports the use of interventions incorporating technology as an important educational tool. This study demonstrated that participation in technology-supported PA during class time is interesting and fun for children, providing a positive influence on their enjoyment of and motivation towards PA. The structure and personal choice of videos, including age-appropriate movements followed by music, animations and dance, influence fun and enjoyment. This finding aligns with the suggestion of Papalia et al. [59] that mobile applications and information technology (IT) based tools such as smartwatches, pedometers, and heart rate monitors increase motivation for PA participation. Tate et al. [60] suggested that these tools are effective in making screen time more active. Furthermore, our results align with Chou et al.’s [61] argument that online-delivered interventions, applications, and mobile games provide low cost, interactivity, and extend reach to subgroups that are typically more challenging to access.

PA engagement in common settings with supportive peers helps maintain enjoyment and participation [54]. Certainly, the use of technology in our study supports the claim of Bonnema et al. [50] that IT provides a higher level of enjoyment, and as Babic et al. [62], Mok et al. [53] and Uzunoz et al. [48] argue, a corresponding enhancement of self-efficacy.

Chinese technology classroom-based intervention is especially important in changing classroom atmosphere. China’s classroom academic atmosphere provides little interaction and is characterized by a traditional pedagogical teaching style (teachers speak, and children listen) [38]. Implementation of Brain Breaks^®^ improves the learning atmosphere. Adding fun and enjoyment results in a more relaxed teaching setting. This application is novel and interesting for Chinese children, but acceptable and easy for teachers to manage. Indeed, the experiences of teachers during the intervention confirm that children were more motivated and engaged when teachers were involved. This engagement is considered an important factor in developing PA adherence habits.

In the present study, no significant difference was detected in learning, self-efficacy and fitness between the experimental and control groups over a three-month classroom-based intervention of Brain Breaks^®^ video exercises. In other words, this study did not demonstrate significant effects on learning, self-efficacy, and fitness as confirmed by other studies. However, in other studies PA intervention did show positive effects on self-efficacy when using video exercises [47,48,49] and holistic learning with video exercises [46,47,48,49,53]. These scholars suggest that structured classroom-based PA and integrated resources provide more than just movement experience and are a source for holistic learning and independence [53]. The lack of such impact in this study is likely the result of the Chinese education system including an overemphasis on academic excellence, traditional pedagogical teaching styles, and lack of culturally–responsive and age-appropriate PA guidelines [35].

At the baseline of the intervention, the experimental and control group have similar achievements at all scales, with slight but statistically not significant differences. Results clearly provide evidence for differences between groups after intervention. Significant group differences were noted for the following scales: (F3) Self-efficacy in learning with video exercises, (F5) Exercise motivation and enjoyment, (F6) Self-confidence on physical fitness, and (F7) Trying to do one’s personal best. Experimental group showed that it steadily increased in these four scales. These results support that PA breaks significantly contributed to the holistic learning of children, and these results concur with the results from other similar studies [4,12,46,47,48,49].

Children demonstrated enhanced self-efficacy in learning, motivation and enjoyment during active breaks which positively affected their perception of personal fitness and their ability to strive towards achieving their personal best. Furthermore, children’s self-confidence improved, and their personal performance motivated subjects to strive for advancement (goal orientation), all of which are associated with the intervention.

For comparation, similar studies from Turkey [48] and Macedonia [47] reported improvement in all APAS scores in intervention and control groups with greater gains reported for the experimental group. No changes in the control group were noted in Poland [46] while in Lithuania [49] lower scores for the control group were found.

To achieve an increased impact on holistic learning in a Chinese setting, several adaptations to classroom-based PA intervention are suggested. These modifications would focus on developing interventions that are culturally specific and better suited to the Chinese education setting. For example, culturally responsive adaptations could include videos using traditional Chinese sports and activities such as Tai Ji Quan movements. These modifications have a two-fold effect. First, content knowledge and activities are more acceptable, familiar, and relevant to Chinese children and teachers. Second, traditional Chinese activities highlighting cultural customs and practices can be uploaded on the Brain Breaks^®^ platform and shared with the rest of the world and would serve to foster better cultural diversity understanding.

The lack of significant improvement in self-efficacy associated with the video exercises and fitness (as compared to the improvement in self-efficacy for students not using the video exercises and fitness) are likely attributed to orthodox practices within the Chinese national education system. During the experiment, the selection of video exercises was completed by teachers, and children had little direct access when selecting videos. Teacher dominated video selection was due to traditional practice, classroom space, student numbers, safety, and ensuring minimal disruption to normal curriculum learning. These practices, we argue, diminished important learning opportunities for children, and as a consequence the intervention did not affect self-efficacy when using video exercises.

Similarly, no differential improvement in personal fitness between children using and not using video exercises was found in this study. The assortment of video exercises from which to select included low-intensity PA such as stretching exercises, low-intensity dances, and easy to perform movements. The duration of these activities was a maximum of 5 min per day [47,53]. No improvement in personal fitness is expected when consideration is given to a short three-month intervention, short PA duration, and low PA intensity.

With such considerations in mind, recent studies suggest that interpreting key principles of high-intensity interval training (HIIT) into incidental PA patterns across the day have shown remarkably consistent health and fitness benefits irrespective of the number of various PA repetitions, duration, and intensity protocols [63]. Incorporating brief sessions of relatively intense PA into daily routine provides numerous practical and health advantages [64]. Adapting classroom-based intervention PA videos in this way by using activities with higher intensity and easy to perform movements, has a greater positive impact on fitness and health.

Effective implementation of classroom-based PA intervention is closely related to the cooperation and interaction of children and teachers during the intervention [54]. Teacher behavior plays an important role in children’s daily PA patterns [65] as does the child’s motivation and knowledge regarding the intervention’s advantages and benefits. Teachers using classroom-based PA interventions are probably generating new ideas on how to implement classroom-based PA [66]. The effectiveness of the application of Brain Breaks^®^ intervention is highly dependent on the teacher’s creativity, personal motivation, technological skills, and flexibility in implementing classroom-based PA interventions into everyday teaching routines [66,67]. Teachers should receive, as part of their academic training, methods for incorporating PA into a classroom setting [46,47]. Classroom-based PA intervention activities are easy to manage, are enjoyable, require only a small amount of additional teacher preparation time, and result in positive outcomes [53]. Teachers using classroom-based PA interventions find enhanced ability to maintain positive classroom behavior, and better student concentration and attention [67]. Previous findings support the incorporation of monitored teacher behaviors into this study. With such monitoring, best pedagogy practice would have ensured integration of teacher monitoring into the intervention and would have maximized the scientific and technological approach to the experimental design.

Brain Breaks^®^ is a product of science and technology promoting the integration of the internet into classroom education to keep pace with contemporary times. The Brain Breaks^®^ physical activity solution also aids in the teaching and learning processes. It aids teaching by improving on task behavior and focus [65,66], adding fun and enjoyment in the teaching setting [48,50,53], resulting in a relaxed teaching atmosphere and the creation of positive classroom behaviors. These facilitate holistic learning through the use of Brain Breaks^®^ videos [46,47,48,49]. Yet teacher and educational administrators should remain cognizant of the following suggestions. To promote the overall development of children, teachers and administrators must remain aware of the difficulty of movement or PA patterns and routines when arranging the content of classroom-based PA intervention. For example, in one video, movements may change from simple to complex while difficulty increases linearly. In a different video setting, a modular video series can achieve a transition from a simple video on Monday to a difficult video by Friday. Teachers should observe children’s performance during classroom-based PA, select the children who performed well and provide them with simple motivational rewards.

What is apparent from this study is that, when applied as a teaching strategy, classroom-based PA intervention activities enhance academic learning while children learn new movements. A second consideration is that different sport-based videos can be used for learning different sport techniques. A third consideration is that observations from this study suggest that improvement in Brain Breaks^®^ videos are possible. Specific modifications could include making videos attractive for people across the life span, particularly for adolescents and even elderly groups, considering the positive effects that classroom-based PA intervention videos had on motivation and improvement of PA participation in type-2 diabetes patients [55]. Hidrus et al. [55] found that special video segments can be developed to promote PA in people with specific health conditions.

The main limitation of this study is the self-reporting nature of the questionnaires. Objective measures for the assessment of children’s PA levels using accelerometers, pedometers, or HR monitors to quantify the amount of PA being completed would have made this study design stronger. Limited time for intervention, strict curricula requirements, limited classroom space, high dependence on the teachers’ experience, teacher creativity, and teacher personal motivation are also limiting factors. However, notwithstanding these limitations, this study is the first to evaluate the effects of Brain Breaks^®^ on Chinese primary school children. This acknowledgement, as well as the experimental design of the study and its realization in a real-world classroom setting, are unique strengths. An additional unique feature is the focus placed on classroom-based PA, and the effect of classroom-based PA on learning, motivation, and self-confidence. Study findings provide strong supporting evidence for potential use of classroom-based PA and knowledge of technological solutions that increase people’s PA engagement. These findings support the need for future studies utilizing large diverse populations to further understand the use and application of these findings.

## 5. Conclusions

The present study confirms that classroom-based and technology-supported PA interventions like Brain Breaks^®^ Physical Activity Solutions provide positive impact on promoting holistic health and children’s attitudes towards PA as manifested by significant improvements in their understanding of the importance of regular PA and exercise, exercise motivation, enjoyment, and striving to enhance personal performance. Improved attitude toward PA based on self-awareness of the importance of PA and increased motivation for participation in PA are important for the overall health and wellbeing of primary school children and are a very important contribution of the Brain Break intervention. Furthermore, the main value of this intervention is not only in providing classroom-based PA, but in effecting holistic learning and personal development of children while performing PA. Participating in video exercises, children take active roles that have positive impact on their personal development by improving personal abilities through movement and striving to do their personal best. Emotional and social components are also improved as children experience enjoyment and fun. Classroom interventions focusing on the use of PA interventions for holistic learning provide an effective strategy supporting teachers’ classroom work. Suggested intervention is easy to facilitate and to integrate in the school day and classroom routine. It provides a variety of possibilities for teachers to use for holistic learning, establishing cross-subject correlations having movement in their bases. In this regard, highlighting the role of teachers, their competences, and motivation, as well as their relationships with children, is an important finding emerging from this study.

## Figures and Tables

**Table 1:** General Characteristics (Mean, Standard Deviation and Frequency) of the Participants.

Variables	Total *n* = 704	EG *n* = 353 (50.1%)	CG *n* = 351 (49.9%)
Age (years)	9.42 ± 0.921	9.41 ± 0.91	9.44 ± 0.92
Gender			
Male	370 (52.6%)	190 (53.8%)	180 (51.3%)
Female	334 (47.4%)	163 (46.2%)	171 (48.7%)
Grade level			
Grade 3	241 (34.2%)	120 (34.0%)	121 (34.4%)
Grade 4	231 (32.8%)	115 (32.6%)	116 (33.1%)
Grade 5	232 (33.0%)	118 (33.4%)	114 (32.5%)

EG = Experimental Group, CG = Control Group.

**Table 2:** Reliability Analysis of Chinese Version of the Attitudes toward Physical Activity Scale (APAS).

	Cronbach’s Alpha
Promoting the Holistic Health (F1)	0.76
Importance of Exercise Habit (F2)	0.65
Self-efficacy in Learning with Video Exercises (F3)	0.76
Self-efficacy in Using Video Exercises (F4)	0.66
Exercise Motivation and Enjoyment (F5)	0.74
Self-confidence on Physical Fitness (F6)	0.69
Trying to do Personal Best (F7)	0.67

**Table 3:** Descriptive Statistics and ANOVA Results Before and After Intervention (pre-test vs. post-test) in Experimental Group (*n* = 353) and Control Group (*n* = 351).

Variables on Physical Activity	Group	Pre-Test	Post-Test	Time			Group			Time * Group		
		Mean (SD)	Mean (SD)	*F*	*p*	*η* ^2^	*F*	*p*	*η* ^2^	*F*	*p*	*η* ^2^
(1)	(2)	(3)	(4)	(5)	(6)	(7)	(8)	(9)	(10)	(11)	(12)	(13)
Benefits (F1)	Con	2.70 (0.58)	3.03 (0.49)	80.15	<0.001	0.10	3.81	0.051	0.01	3.96	0.047	0.01
	Exp	2.69 (0.63)	2.91 (0.65)									
Importance (F2)	Con	3.08 (0.50)	3.15 (0.49)	37.38	<0.001	0.05	0.30	0.581	<0.01	12.21	0.001	0.02
	Exp	2.99 (0.49)	3.26 (0.53)									
Learning (F3)	Con	2.34 (0.48)	2.56 (0.56)	47.70	<0.001	0.06	6.82	0.009	<0.01	0.02	0.886	0.01
	Exp	2.42 (0.63)	2.65 (0.77)									
Self-efficacy (F4)	Con	3.09 (0.55)	3.20 (0.46)	6.44	0.011	0.01	0.12	0.735	<0.01	1.85	0.174	0.01
	Exp	3.14 (0.61)	3.17 (0.58)									
Fun (F5)	Con	3.03 (0.56)	3.14 (0.41)	45.36	<0.001	0.06	7.38	0.007	0.01	7.11	0.008	0.01
	Exp	3.04 (0.54)	3.28 (0.50)									
Fitness (F6)	Con	2.85 (0.49)	3.10 (0.38)	163.73	<0.001	0.19	12.83	<0.001	0.02	2.63	0.105	0.01
	Exp	2.90 (0.48)	3.23 (0.47)									
Personal Best (F7)	Con	2.93 (0.44)	3.15 (0.46)	150.36	<0.001	0.18	23.48	<0.001	0.03	11.26	0.001	0.02
	Exp	2.99 (0.50)	3.36 (0.54)									

Con = Control, Exp = Experimental.
